# Unpacking the Association between Family Functionality and Psychological Distress among Chinese Left-Behind Children: The Mediating Role of Social Support and Internet Addiction

**DOI:** 10.3390/ijerph192013327

**Published:** 2022-10-15

**Authors:** Xiaoyan Fan

**Affiliations:** School of Social Development, East China Normal University, Shanghai 200241, China; 52203500002@stu.ecnu.edu.cn ; Tel.: +86-13391336985

**Keywords:** family functionality, psychological distress, social support, Internet addiction, left-behind children

## Abstract

Family functionality has been proven to be associated with adolescents’ and children’s mental health, but its indirect mechanisms among left-behind children have rarely been investigated in the Chinese context. This study aims to explore the direct effect of family functionality on psychological distress and the mediating roles of social support and Internet addiction among Chinese left-behind children. Based on multistage random sampling, 1355 students were recruited to participate in a school-based questionnaire survey in Jiangsu Province. Structural equation modeling using Amos 26.0 was used to test the direct and indirect paths of the variables. The results support our hypotheses, suggesting that family functionality has a direct effect on the psychological distress of left-behind children. Meanwhile, the association between family functionality and psychological distress is mediated by social support and Internet addiction, separately and sequentially. The findings suggest that effective social work interventions for psychological distress should be targeted toward social support and Internet addiction among left-behind children.

## 1. Introduction

With the continuous development of industrialization and urbanization, a large number of young and middle-aged rural laborers have flocked to cities for employment [1]. However, due to the dualistic social structure between urban and rural areas, especially the institutional restrictions concerning education and social security, migrants must leave their children behind in rural areas [2]. This scenario gives rise to the problem of left-behind children, who are rural household youth under 16 years old, with both or one parents away, and the remaining parents having no legal guardianship over the child/children [3]. According to the data released by the Ministry of Civil Affairs, Chinese rural areas retained 6.44 million left-behind children at the end of 2020 [4]. A series of social problems arising from such a huge group have attracted widespread attention from policymakers and researchers. With the increase in family income due to parents’ working away from home, the material conditions and basic lifestyle of left-behind children are guaranteed. However, the alienation of the parent–child relationship, children’s online game addictions, and mental health problems caused by parental absence must not be ignored.

According to data from the World Health Organization (WHO), the global prevalence of mental disorders among children and adolescents ranges from 12% to 28%, and these numbers are currently on the rise. Meanwhile, data from the Institute of Psychology of the Chinese Academy of Sciences show that the rate of depression among Chinese adolescents and children in 2020 was 24.6%, making this disorder a major threat to their healthy growth. Psychological distress is widely used to describe mental health conditions, including symptoms such as anxiety and depression. Among children, those that are left-behind have a higher likelihood of experiencing psychological distress and behavioral problems than their peers [5]. For left-behind children, parental absence is an inevitable inconvenience and often leads to family functioning problems. Numerous studies have investigated this issue, determining that family functionality is an important predictor of left-behind children’s psychological distress [6]. Children living in a caring family can have a more positive attitude toward life, participate more actively in social activities, and attain higher academic achievement [7]. In contrast, family dysfunction is associated with psychological and behavioral problems in children. In addition, for left-behind children, prolonged separation from parents can lead to changes in family functioning and reduced parent-child communication and interaction, which can then lead to psychological distress such as anxiety, depression, and loneliness [6,8].

As a special population group in mainland China, left-behind children and their healthy development are affected by the degradation of family function due to long-term separation from parents; thus, they face problems such as psychological disorders. In such a context, this study aims to investigate the direct effect of family functionality on the psychological distress of left-behind children and also to test the indirect effect mechanisms of social support and Internet addiction. The findings can shed light on social policies and social work interventions to enhance the psychological well-being of left-behind children.

## 2. Literature Review

### 2.1. Family Functionality and Psychological Distress

As an important setting for children’s socialization, the family plays a vital role in promoting children’s mental well-being [7]. Family functionality is a dynamic concept that refers to the family members fulfilling their roles, strengthening cohesion, solving issues, and communicating positively [6,9]. Adaptability and cohesion are the two main dimensions of family functionality [10]. The family resilience theory [11] states that while encountering stress, crisis, or challenges, people living in families with positive functioning can communicate, interact, share ideas, solve problems together, and even transform crises into opportunities [6,11]. During this process, the family can become more adaptable to the environment and develop new abilities such that the children can gain confidence and a sense of security [12]. High family functionality helps individuals adapt effectively and reduce negative emotions. High levels of cohesion are associated with low levels of anxiety and depression [6]. Meanwhile, effective family communication improves the parent–child relationship, makes children feel more support, and helps solve their challenges in growing up such that psychological distress can be prevented [12].

The parents’ migration—characterized by long-term separation and a low of frequency interaction with their children—changes the traditional family functionality [6,13]; consequently, these families do not have enough ability to modify their structure to adapt to the environment when facing pressure [7]. Various studies reveal that parental absence eliminates emotional connection, leads to disengagement, and impairs family functionality, subsequently increasing the risk of left-behind children experiencing psychological distress [14,15]. Overall, based on the above findings, the following hypothesis is proposed.

**H1:**  *Family functionality is negatively related to left-behind children’s psychological distress.*

### 2.2. Family Functionality, Social Support, and Psychological Distress

According to the social support theory, social support is the capacity of a social network to offer psychological and material resources to help individuals deal with stress [16]. For children, their social support mainly comes from their family, school, and peers [17]. As one of the essential elements in children’s development, adequate social support can provide sufficient assistance, meet their demands, reinforce their abilities, and reduce their psychological distress. Perceived high levels of social support are associated with fewer psychological problems in children [18]. Children with rich social networks are more stable and positive in coping with pressure and have fewer behavior problems than their peers. However, left-behind children have more difficulties in obtaining resources in many dimensions. The separation from their parents impairs the care and warmth that left-behind children usually obtain from their families [6,7]. These children may also communicate rarely with their peers or teachers in school, and are thus easily neglected. Moreover, school bullying occurs more frequently toward left-behind children compared with non-left-behind children [19]. For example, using data from 1507 adolescents, Chang et al. suggested that parental and peer social support are directly correlated to depression [18]. Duru and Balkis found that support from teachers and classmates at school can reduce the risk of adolescents’ psychological distress [20].

Family functionality has a positive and significant association with children’s social support. Families with high cohesion and adaptability can provide more social support for children [6,11]. Children with positive family functionality can feel more warmth and care from their parents. These children can also interact more positively with their teachers and peers, thus receiving more support and encouragement that can significantly protect children from psychological distress and promote their psychological well-being [21,22]. For left-behind children, parental absence weakens their emotional bonds. Long-term separation from their parents also leads to reduced family cohesion and adaptation. Furthermore, left-behind children with low levels of family functionality lack interaction with their parents, teachers, and peers, and thus may obtain less social support [23,24,25]. Therefore, we propose the following hypothesis.

**H2:**  *Social support mediates the relationship between family functionality and left-behind children’s psychological distress.*

### 2.3. Family Functionality, Internet Addiction, and Psychological Distress

With the popularity of the Internet, the online addiction of children has become a severe problem. Normally, Internet addiction denotes spending excessive time online and causes detrimental effects on an individual’s psychological and social development. In particular, left-behind children are at higher risk for Internet addiction than non-left-behind children [26]. Theoretical and empirical findings reveal the correlation between family functionality and children’s Internet addiction. According to the cognitive–behavioral model [27], problems caused by the lack of beneficial parenting and intimate parent–child relationship prompt individuals to escape the real world and seek more positive responses from the Internet [28]. For left-behind children, their unusual family functionality and growing experience influence their psychology and behaviors [29]. Given their lack of parental care and attention, left-behind children often become addicted to the virtual world, which causes problematic Internet use behaviors. The inadequate parental supervision also allows left-behind children to have more and higher frequency of Internet use, which may lead to addiction [26,30].

In turn, Internet addiction can lead to children’s psychological distress and problematic behaviors [31]. While using the Internet to escape harmful family influences, the problematic use can also increase a child’s anxiety, depression, and other mental symptoms [32,33]. For example, Guo found that Internet addiction can increase the risk of depression in left-behind children [26]. Using data from 2196 students, Arrivillaga and Extremera demonstrated that students who overuse the Internet report psychological maladjustment [34]. Therefore, family functionality can affect left-behind children’s Internet addiction, which in turn can influence their psychological distress. Thus, the following hypothesis is proposed.

**H3:**  *Internet addiction mediates the relationship between family functionality and left-behind children’s psychological distress.*

### 2.4. Social Support and Internet Addiction

The correlation between social support and children’s Internet addiction has also been investigated [35]. A positive family interaction and secure attachment can alleviate Internet abuse among adolescents by providing them with adequate warmth and diminishing their anxiety and depression [36]. In addition to their family, children perceive greater interaction and support from their teachers and peers, which prompts them to keep in touch with others and avoid problematic Internet use [23,24,37]. In contrast, inadequate social support may lead to a high risk of adolescent Internet addiction [36]. Low levels of parental monitoring and high levels of emotional neglect may increase the risk of Internet addiction in left-behind children. Similarly, left-behind children with poor teacher–student and peer relationships or who suffer from peer exclusion are more likely to develop Internet addiction [38].

However, other studies also found a bidirectional relationship between social support and Internet addiction [39]. The social displacement theory [40] suggests that due to the excessive time spent in the virtual online world, children have to sacrifice social interactions with family members and friends, which leads to a lack of social support from these significant others [41]. Based on the abovementioned findings, the following hypothesis is proposed.

**H4:**  *Left-behind children’s social support negatively predicts Internet addiction, and the two variables play serial mediating roles in the association between family functionality and psychological distress.*

### 2.5. The Knowledge Gap and Theoretical Framework

**Figure 1:**
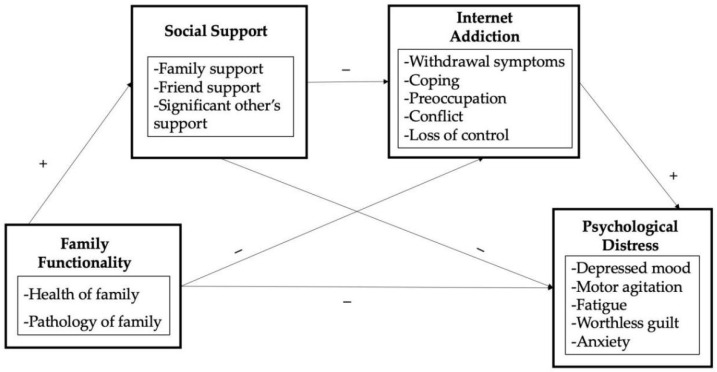
The theoretical framework.

The literature review revealed several knowledge gaps regarding family functionality and the psychological disorders of left-behind children. Although family functionality has been proven to correlate with the mental health of both adolescents and children, the indirect mechanisms regarding left-behind children are rarely investigated in the Chinese context. The effect of social support and Internet addiction on the relationship has been determined, but the chain mediating effect still requires further exploration. Therefore, on the basis of the family resilience theory, the social support theory, and the cognitive–behavioral model, this study constructs an integrated theoretical framework (Figure 1) to investigate the direct and indirect effects of family functionality on the psychological distress of left-behind children in the Chinese context.

## 3. Methods

### 3.1. Participants

Based on a multistage random sampling, the participants in this study included 1355 middle and high school students in Jiangsu Province. The sample was randomly selected, mainly from 24 classes in 4 junior high schools and 24 classes in 4 high schools. In Table 1, the demographic characteristics of the sample are presented. Among the sample, 42.9% (581) were males and 57.1% (774) were females. As for their household registration, 44.3% (600) had a rural household registration, and 55.7% (755) had an urban household registration. The age of the participants ranged from 10 to 19 years old. A total of 43.8% of the participants came from families with only one child, and 13.8% came from single-parent families. With respect to the educational level of their parents, 35.3% of the participants’ fathers had a middle school diploma, followed by a high school diploma (29.1%), and a vocational school diploma (12.2%). A total of 37.7% of the participants’ mothers had a middle school diploma, followed by a high school diploma (20.7%), and an elementary school diploma (13%).

### 3.2. Procedure

The data collection of this study was mainly conducted in the schools. First, we contacted the sampled schools through the local education department and explained the contents and arrangements of this research in order to obtain their permission and cooperation. Second, before administering the questionnaires, we explained the purpose of this study and the research questions to the sampled students and distributed informed consent forms to the students and their guardians. Third, using the students’ extracurricular time, the researcher and research assistants administered the survey to the volunteered students in the classrooms, with both the students and their guardians signing the informed consent forms. Before students filled out the questionnaire, instructions and precautions for filling out the questionnaire were explained, and students were told that they could give up at any time during the process. Fourth, after the questionnaires were completed, the research assistants checked and collected the questionnaires. The participants were all volunteers, and the students filled out the questionnaires anonymously. The research process strictly followed research ethics and was approved by the university committee on human research protection of the researcher’s university.

### 3.3. Measurements

#### 3.3.1. Family Functionality

The family functionality of left-behind children was evaluated using the general functioning subscale of the family assessment device (FAD) [42]. The FAD’s 12-item short form is answered using a 4-point Likert scale of 1 (totally disagree) to 4 (strongly agree). This scale contains two main dimensions: health and pathology of the family. The mean scores of the two dimensions were calculated separately. Higher average scores indicate that the participants have higher levels of family functionality. In the current study, the shorter version scale showed good internal consistency (Cronbach’s alpha = 0.864).

#### 3.3.2. Psychological Distress

The 10-item Kessler psychological distress scale was used to assess participants’ psychological distress [43]. Left-behind children were asked about their recent emotions in the last month. This measure has five components, including depressed mood, motor agitation, fatigue, worthless guilt, and anxiety. It is graded on a 5-point Likert scale of 1 (never) to 5 (always). The items of each dimension were summed, and the mean value represents the score of each certain dimension. A higher score suggests that the left-behind children are experiencing more psychological distress. In this investigation, the scale’s Cronbach’s alpha was 0.952.

#### 3.3.3. Social Support

Left-behind children’s perceptions of social support was tested with the multidimensional scale of perceived social support [44]. The 12-item MSPSS is scored on a 7-point Likert scale of 1 (completely disagree) to 7 (completely agree). We calculated the mean scores of each of the three dimensions (family, friend, and significant other’s social support) to indicate the degree of social support perceived by the left-behind children. Higher average scores indicate that the participants have a higher levels of perceived social support. In the current study, the MSPSS scale displayed very high internal consistency (Cronbach’s alpha = 0.946).

#### 3.3.4. Internet Addiction

The compulsive Internet use scale (CIUS) was used to measure the participants’ Internet addiction [45]. The CIUS consists of 12 items, each of which is assessed on a 5-point Likert scale of 1 (never) to 5 (always). This measure has five components, including loss of control, conflict, preoccupation, coping, and withdrawal symptoms. The items of each dimension were summed to calculate the mean value to indicate the score of the dimension. A higher score revealed that the left-behind children had a higher level of Internet addiction. The internal consistency in this study was acceptable (Cronbach’s alpha = 0.860).

#### 3.3.5. Control Variables

The main socio-demographic control variables in this study were gender, household registration, and whether or not they were single-parent families. Gender (male = 0, female = 1), household registration (rural = 0, urban = 1), and single-parent family (no = 0, yes = 1) were all self-administered questions.

### 3.4. Analytical Methods

In this study, SPSS 26.0 was adopted to conduct the statistical analyses, while Amos 26.0 was used to verify the direct and indirect paths of the variables. First, we calculated the frequencies, percentages, means, and standard deviations of the socio-demographic variables to describe their characteristics. Second, to verify the direct and indirect effects, the structural equation model of Amos 26.0 was used. In regard to the measurement model, we combined all the four scales together and performed confirmatory factor analysis (CFA) on all constructs at once, verifying the fitness of the observed variables to the four latent variables. As for the structural model, we tested the direct effect of family functionality on the psychological distress of left-behind children and the chain mediating effect of social support and Internet addiction. In this study, the CFI and RMSEA are utilized to verify the model fit of the structural and measurement model. CFI above 0.9 and RMSEA less than 0.08 suggest that the data fit well with the hypothesized model.

## 4. Results

### 4.1. Measurement Model

This study utilized confirmatory factor analysis (CFA) to verify the measurement model. The results suggest that the measurement model provides good fit indices: χ^2^ = 532.931, df = 84, *p* < 0.001, CFI = 0.928 > 0.9, and RMSEA = 0.073 < 0.08. The four latent variables (family functionality, social support, Internet addiction, psychological distress) in the measurement model were well represented by the observed variables, and all the factor loadings were significant at the 0.001 level. Factor loadings ranged from 0.800 to 0.835 for family functionality, from 0.755 to 0.929 for social support, from 0.471 to 0.849 for Internet addiction, and from 0.812 to 0.957 for psychological distress (shown in Table 2).

### 4.2. Structural Model

**Figure 2:**
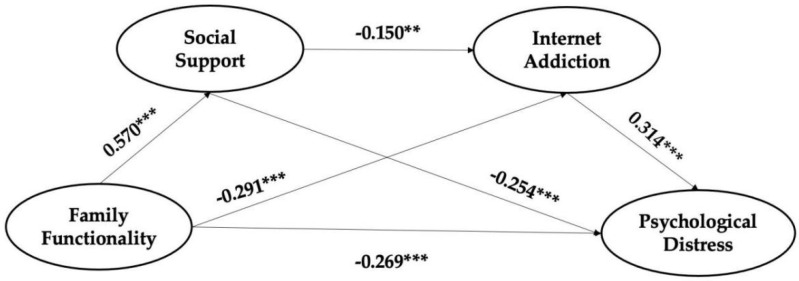
The structural model (** *p* < 0.01; *** *p* < 0.001).

The results showed a good fit to the data: χ^2^ = 536.032, df = 122, *p* < 0.001, CFI = 0.935, and RMSEA = 0.074. Figure 2 and Table 3 showed the standardized coefficient of variables in the structural model. As shown in Figure 2, family functionality was directly and positively correlated with social support (β = 0.570, *p* < 0.001) and negatively associated with psychological distress (β = −0.269, *p* < 0.001) and Internet addiction (β = −0.291, *p* < 0.001). Moreover, family functionality was indirectly and significantly correlated to left-behind children’s psychological distress through the mediating role of social support and Internet addiction (β = 0.314, *p* < 0.001 for the association between Internet addiction and psychological distress; β = −0.254, *p* < 0.001 for the association between social support and psychological distress). Moreover, there was a chain mediating effect between family functionality and psychological distress (β = −0.150, *p* < 0.01 for the association between social support and Internet addiction). In Table 3, we present the standardized path coefficient for the structural model.

Among all the control variables, only gender (β = 0.131, *p* < 0.001) has a significant impact on left-behind children’s psychological distress. This result suggests that the female left-behind children have higher levels of psychological distress than the males.

## 5. Discussion

Based on a sample of left-behind children, this study investigates the direct and indirect effects of family functionality on the psychological distress of left-behind children. The findings have important theoretical and practical implications.

### 5.1. The Effect of Family Functionality on Psychological Distress

Family functionality has a significant, direct, and negative effect on the psychological distress of Chinese left-behind children; thus, Hypothesis 1 is verified. This means that left-behind children with negative family functionality have high psychological distress levels. At the same time, this finding is consistent with the family resilience theory [11], which proposes that good family functionality—including a warm family atmosphere, positive adaptation and cohesion, and a good parent–child relationship—can prevent the negative effects of crises and stress on family members [6,10]. Specifically, one of the important roles of family functionality is to increase the cohesion within the family and to promote the members’ well-being. Thus, positive family functioning promotes interaction and communication among family members and helps the children to gain confidence and security, thereby reducing the probability of psychological distress and improving their mental health. One possible explanation for the findings is that the family is a crucial setting for children’s healthy development, and it plays an important role in their development and socialization. Family functioning has various manifestations, including not only an economic function, but also nurturing and educational functions. As such, good family functionality can have a significant impact on children’s education, development, and mental health [13]. For the subject of this study, Chinese left-behind children have their own special characteristics. In search of better economic conditions, a large number of migrant workers move to cities to work, but their children are forced to stay and live in their hometowns due to urban–rural restrictions. Given that parents are busy with their work, they do not have enough time to go home to visit their children. The lack of interaction and communication between parents and their children causes the left-behind children to receive less care and attention, such that their family functioning conditions are relatively lower than those of non-left-behind children. This scenario can have severe negative influences on their psychosocial development. In summary, negative family functioning leads to psychological dysfunction; the poorer the family functioning, the greater the impact on psychological distress.

Meanwhile, although most of the empirical findings confirm the impact of family functionality on the psychological distress of left-behind children, a few studies found no significant effects of family functioning on psychological factors (e.g., anxiety, depression) among left-behind children [14,15]. However, the current findings are in line with most of the empirical studies, resolving the academic controversy over this relationship on left-behind children by concluding that family functionality has a significant direct effect on the psychological distress of left-behind children and confirming the importance of family in their growth and development.

### 5.2. The Mediating Effects of Social Support and Internet Addiction

The findings demonstrate that social support mediates the relationship between family functioning and the psychological distress of left-behind children, thereby confirming Hypothesis 2. Namely, left-behind children with low levels of family functionality, which can lead to their low levels of social support, have high probabilities of experiencing psychological distress. This finding is consistent with the social support theory [16] and previous empirical findings [20]. That is, social support is a mediator of the effect of family functionality on psychological distress in left-behind children. Positive family functionality enables children to receive more social support from parents, teachers, and peers, which in turn can reduce the occurrence of psychological problems and improve their well-being [22,23,25]. In this study, the parents are mostly working away from home and have limited contact time with their left-behind children, and parent–child communication is lacking. Thus, their level of family functionality is lower than that of their peers. Left-behind children feel low levels of parental care, teacher–student interaction, and peer support, which can lead to emotional disorders, such as anxiety, depression, and psychological distress. Therefore, the special characteristics of left-behind children can lead to their lower level of family functioning and social support, and thus they have more severe psychological problems and a higher likelihood of experiencing psychological distress than non-left-behind children.

This study also finds that family functionality can affect left-behind children’s psychological distress through the mediating effect of Internet addiction. Thus, Hypothesis 3 is also verified. That is, when left-behind children have low levels of family functionality, which can lead to their high levels of Internet addiction, then they may also experience high psychological distress. This finding is consistent with the cognitive–behavioral model and the results of most of the previous empirical studies [27,28]. Given the lack in good parenting, parental care, and positive parent–child relationships, left-behind children spend most of their time online, which is more likely to lead to Internet addiction over time [26,30]. Internet addiction can have severe negative effects on left-behind children’s educational outcome, psychological well-being, and behavioral development [32,33]. In rural areas of mainland China, parents have to move to cities for work, and left-behind children usually live with their grandparents. However, grandparents take care of left-behind children by providing more of basic material things such as food and drink, but lack care for mental health and behavioral development. In the Internet era, the development of technology is a double-edged sword in the lives of left-behind children. On the one hand, left-behind children can make full use of the advantages of the Internet for their own learning, to gain knowledge, and to broaden their horizons. On the other hand, left-behind children who are neglected tend to indulge in cell phone usage, video viewing, and online games. Prolonged problematic Internet use can lead to psychological disorders, such as anxiety and depression, and to behavioral problems, such as aggression and cyber-bullying. Thus, the influence of family functionality on the psychological distress of left-behind children can also be realized through the indirect effect of Internet addiction.

In addition, an important finding of this study is that social support and Internet addiction can also play a chain mediating role on the effect of family functionality on the psychological distress of left-behind children. Thus, Hypothesis 4 is confirmed. This finding is also consistent with previous research showing that that left-behind children who receive less parental love and care also have less social support from their family, school, and peers. Inadequate social support causes left-behind children to spend more time on cell phones and computers, which can lead to Internet addictive behaviors [35,46]. Long-term Internet addiction not only affects the academic performance of left-behind children, but also their mental health [36,37]. At the same time, this study further verifies the causal association of social support and Internet addiction, confirming that the social displacement theory is not applicable to this sample of left-behind children.

## 6. Implications

On the basis of the family resilience theory, the social support theory, and the cognitive-behavioral model, this study forms an integrated theoretical framework to investigate the direct effects of family functionality on the psychological distress of left-behind children and the indirect effects of social support and Internet addiction. This study has important theoretical and practical implications. At the theoretical level, the findings not only verify the applicability of the related theories to the Chinese cultural contexts, especially to the special group of left-behind children, but also further expand the scope and the explanation of the theories [11,16,27]. At the same time, the chain mediating model of family functionality on the psychological distress of left-behind children also provides a theoretical framework for subsequent studies. At the practical level, the findings provide guidelines for interventions regarding psychological and behavioral problems. Family functionality and social support have significant effects on both the behavior and psychology of left-behind children, and thus reducing Internet addiction and psychological disorders in children requires more family care, good parent–child relationships, and greater social support. Therefore, future social work interventions for left-behind children must advocate for more care and spiritual support from parents and more social support from families, schools, and peers in order to reduce their behavioral problems and improve their psychological well-being [47,48].

## 7. Limitations

This study also has several limitations. First, the data are derived from self-administered questionnaire reports completed by left-behind children, which lack responses from significant others, such as parents and teachers, resulting in relatively sensitive variables (e.g., Internet addiction) that may be inaccurate. In the future, increasing the acquisition of data from multiple subjects can be considered to accurately reflect the psychological and behavioral problems of left-behind children. Second, this study is a cross-sectional design, which can only reveal the correlation between variables. Therefore, the causal mechanism needs to be further tested by longitudinal data in future studies. Third, the data are only collected from one province in mainland China, and future research can collect data in different provinces to improve the representativeness of the sample. Fourth, Internet addiction and psychological disorders among left-behind children may be influenced by other factors, such as family socioeconomic status, community environment, resilience, and personality traits. These factors need to be incorporated into the theoretical framework to comprehensively investigate the mechanisms of behavioral and psychological problems among left-behind children.

## 8. Conclusions

The findings reveal that family functionality can have a significant direct effect on the psychological distress of left-behind children. Moreover, the associations can be separately and sequentially mediated by social support and Internet addiction. Effective social work interventions for psychological distress must therefore target social support and Internet addiction among Chinese left-behind children.

## Figures and Tables

**Table 1:** Demographic characteristics of the sample.

Variable		Total Sample (*n* = 1355)
Age (Years)		Mean = 15.0 (S.D. = 1.83)
Range 10–19		
Gender	Male	581 (42.9%)
	Female	774 (57.1%)
Household Registration	Rural	600 (44.3%)
	Urban	755 (55.7%)
Only one child family	Yes	593 (43.8%)
	No	762 (56.2%)
Single-parent family	Yes	187 (13.8%)
	No	1168 (86.2%)
Father’s education level	Illiterate	26 (1.9%)
	Primary school diploma	86 (6.3%)
	Middle school diploma	477 (35.3%)
	High school diploma	394 (29.1%)
	Vocational school diploma	165 (12.2%)
	Junior college diploma	121 (8.9%)
	University or higher	86 (6.3%)
Mother’s education level	Illiterate	95 (7.0%)
	Primary school diploma	176 (13.0%)
	Middle school diploma	510 (37.7%)
	High school diploma	281 (20.7%)
	Vocational school diploma	130 (9.6%)
	Junior college diploma	101 (7.5%)
	University or higher	62 (4.5%)

**Table 2:** Results of measurement model.

Latent Variables	Observed Variables	Factor Loadings
Family functionality	Health of the family	0.835 ***
	Pathology of the family	0.800 ***
Social support	Family support	0.755 ***
	Friend support	0.890 ***
	Significant other’s support	0.929 ***
Internet addiction	Withdrawal symptoms	0.663 ***
	Coping	0.569 ***
	Preoccupation	0.849 ***
	Conflict	0.471 ***
	Loss of control	0.832 ***
Psychological distress	Depressed mood	0.957 ***
	Motor agitation	0.847 ***
	Fatigue	0.879 ***
	Worthless guilt	0.812 ***
	Anxiety	0.821 ***

*** *p* < 0.001.

**Table 3:** Coefficients of path analysis.

Predictor	Outcome	β
Family functionality	Psychological distress	−0.269 ***
Family functionality	Social support	0.570 ***
Family functionality	Internet addiction	−0.291 ***
Social support	Internet addiction	−0.150 **
Internet addiction	Psychological distress	0.314 ***
Social support	Psychological distress	−0.254 ***

** *p* < 0.01; *** *p* < 0.001.

## Data Availability

The data presented in this study are available upon request from the corresponding author.
